# A Novel Circular Rep-Encoding Single-Stranded DNA from Holstein Calves

**DOI:** 10.1128/mra.01192-22

**Published:** 2023-02-27

**Authors:** Stephen F. Marino

**Affiliations:** a German Federal Institute for Risk Assessment, Department of Biological Safety, Berlin, Germany; Montana State University

## Abstract

A novel, circular DNA molecule was identified in the serum of 4-week-old Holstein calves via Illumina sequencing. Comparisons with the NCBI nucleotide data bank indicate that the sequence is unique. The circle contains one predicted open reading frame (ORF), whose translated protein sequence shows high similarity to bacterial Rep proteins.

## ANNOUNCEMENT

Bovine meat and milk factors (BMMFs) are circular DNAs claimed to be involved in the development of cancer and to be present only in European cattle (Bos taurus) breeds (1). Since BMMFs were initially detected in adult cattle, we investigated whether juvenile cattle also harbor these molecules. We obtained blood from 4-week-old Holstein calves from an unrelated study approved and conducted according to the standards and requirements of the animal protection authority of the Free University of Berlin (approval number TZ 0002/21). The samples were collected via arterial puncture, and after clotting, serum was extracted by centrifugation for 20 min at 2,000 × *g*, immediately aliquoted, and frozen at −20°C. Total nucleic acid was isolated from 200 μL serum (High Pure Viral Nucleic Acid Isolation Kit; Roche), and 930 ng were added to a 40-μL rolling-circle amplification (RCA) reaction with φ29 polymerase and random hexamer primers (2) and incubated for 17 h at 30°C. Amplified double-stranded DNA (315 ng) was used to prepare a fragment library for Illumina short-read sequencing. Among the resulting assembled contigs was a non-BMMF, unique, circular DNA resembling members of the CRESS (Circular, Rep-Encoding, Single-Stranded DNA) group of molecules (3).

Fragment libraries were prepared using the Illumina DNA prep (M) Tagmentation Kit with Nextera DNA combinatorial dual (CD) indexes and sequenced using a NextSeq midoutput flow cell (300 cycles) on a NextSeq 550 sequencer. The raw data were quality controlled and trimmed using fastp (4), yielding 3.1 × 10^6^ paired-end reads (mean length, 146.7 bases; range, 15 to 151 bases). The contigs were assembled with Unicycler v0.4.4 (5) utilizing the SPAdes assembly algorithm (6) and automatically flagging circular assemblies. Default software parameters were used.

Annotation was performed using Geneious v2020.2.2. Read mapping identified 75,894 overlapping reads for the unique 1,644-bp circular contig, Kb273, with a positional non-end gap mean coverage of 6,790.5× and a GC content of 41%. The molecule contains one predicted open reading frame (ORF) of 651 bases, whose translated protein sequence via BLASTP (7) shows high similarity to bacterial Rep family proteins, with the closest match being a Rep from Streptococcus dysgalactiae (NCBI protein accession number MSU87956.1) showing 99% sequence coverage and 58% identity. Multiple nucleotide BLAST (8) searches using different strategies failed to return hits for the predicted ORF and returned only insubstantial hits for the rest of the contig, either whole or divided into segments. The predicted Rep protein encoded by Kb273 lacks 50 N-terminal residues present in the S. dysgalactiae Rep (MSU87956.1). However, a three-dimensional structure prediction using RoseTTAFold (9) clearly shows the presence of the Rep HUH motif and tyrosine residues conserved in the catalytic sites of diverse Rep proteins. In the tested group, five of eight calves harbored the Kb273 molecule. Identical processing and sequencing of control samples using water instead of serum yielded neither Kb273 nor similar sequences.

### Data availability.

The sequence of Kb273 has been deposited in GenBank under the accession number OP796838 and the associated raw reads under BioProject accession number PRJNA900058.

## References

[1] Zur Hausen H, Bund T, de Villiers E-M. 2017. Infectious agents in bovine red meat and milk and their potential role in cancer and other chronic diseases. Curr Top Microbiol Immunol 407:83–116. doi:10.1007/82_2017_3. 28349283

[2] Hutchison CA, III, Smith HO, Pfannkoch C, Venter JC. 2005. Cell-free cloning using phi29 DNA polymerase. Proc Natl Acad Sci USA 102:17332–17336. doi:10.1073/pnas.0508809102. 16286637 PMC1283157

[3] Zhao L, Rosario K, Breitbart M, Duffy S. 2019. Eukaryotic circular rep-encoding single-stranded DNA (CRESS DNA) viruses: ubiquitous viruses with small genomes and a diverse host range. Adv Virus Res 103:71–133. doi:10.1016/bs.aivir.2018.10.001. 30635078

[4] Chen S, Zhou Y, Chen Y, Gu J. 2018. Fastp: an ultra-fast all-in-one FASTQ preprocessor. Bioinformatics 34:i884–i890. doi:10.1093/bioinformatics/bty560. 30423086 PMC6129281

[5] Wick RR, Judd LM, Gorrie CL, Holt KE. 2017. Unicycler: resolving bacterial genome assemblies from short and long sequencing reads. PLoS Comput Biol 13:e1005595. doi:10.1371/journal.pcbi.1005595. 28594827 PMC5481147

[6] Bankevich A, Nurk S, Antipov D, Gurevich AA, Dvorkin M, Kulikov AS, Lesin VM, Nikolenko SI, Pham S, Prjibelski AD, Pyshkin AV, Sirotkin AV, Vyahhi N, Tesler G, Alekseyev MA, Pevzner PA. 2012. SPAdes: a new genome assembly algorithm and its applications to single-cell sequencing. J Comput Biol 19:455–477. doi:10.1089/cmb.2012.0021. 22506599 PMC3342519

[7] Gish W, States DJ. 1993. Identification of protein coding regions by database similarity search. Nat Genet 3:266–272. doi:10.1038/ng0393-266. 8485583

[8] Altschul SF, Gish W, Miller W, Myers EW, Lipman DJ. 1990. Basic local alignment search tool. J Mol Biol 215:403–410. doi:10.1016/S0022-2836(05)80360-2. 2231712

[9] Baek M, DiMaio F, Anishchenko I, Dauparas J, Ovchinnikov S, Lee GR, Wang J, Cong Q, Kinch LN, Schaeffer RD, Millán C, Park H, Adams C, Glassman CR, DeGiovanni A, Pereira JH, Rodrigues AV, van Dijk AA, Ebrecht AC, Opperman DJ, Sagmeister T, Buhlheller C, Pavkov-Keller T, Rathinaswamy MK, Dalwadi U, Yip CK, Burke JE, Garcia KC, Grishin NV, Adams PD, Read RJ, Baker D. 2021. Accurate prediction of protein structures and interactions using a three-track neural network. Science 373:871–876. doi:10.1126/science.abj8754. 34282049 PMC7612213

